# Assessing the Effectiveness of Professional Development Training on Autism and Culturally Responsive Practice for Educators and Practitioners in Ethiopia

**DOI:** 10.3389/fpsyt.2020.583674

**Published:** 2021-02-23

**Authors:** Waganesh A. Zeleke, Tammy L. Hughes, Gibbs Kanyongo

**Affiliations:** ^1^Department of Counseling, Psychology and Special Education, Duquesne University, Pittsburgh, PA, United States; ^2^Department of Educational Foundations and Leadership, Duquesne University, Pittsburgh, PA, United States

**Keywords:** Ethiopia, professional development training, multicultural mental health practice, training effectiveness, autism

## Abstract

This study examines the effect of professional development training on educators' and practitioners' knowledge of Autism and the use of culturally responsive practices. Using a single group, pre-post design, data was gathered from 34 educators and health professionals (i.e., teachers, counselors, psychologists, therapists, therapeutic care workers, social workers, and nurses) in Ethiopia. A week-long training covering ASDs and culturally responsive evidence-based training was provided to participants. Results showed significant improvement in participants' knowledge about ASD symptoms, nature, characteristics, as well as intervention selection. Participants' use of culturally informed approaches, in their area of professional service, showed a high level of participants' knowledge and low-level use of culturally responsive practices, policies, and procedures. Recommendations for addressing cultural factors impacting the diagnosis and treatment-seeking approaches to ASD in Africa are provided.

## Introduction

Autism spectrum disorder (ASD) is a developmental and neurological disorder characterized by social and communication deficits as well as restricted, repetitive behavior patterns. Although autism is viewed as a set of criteria, individuals on the spectrum show a range of overactive sensitivities as well as under responsive characteristics. For example, consider this illustration adopted from Johnson (1) and reprinted with permission.

**Table:** 

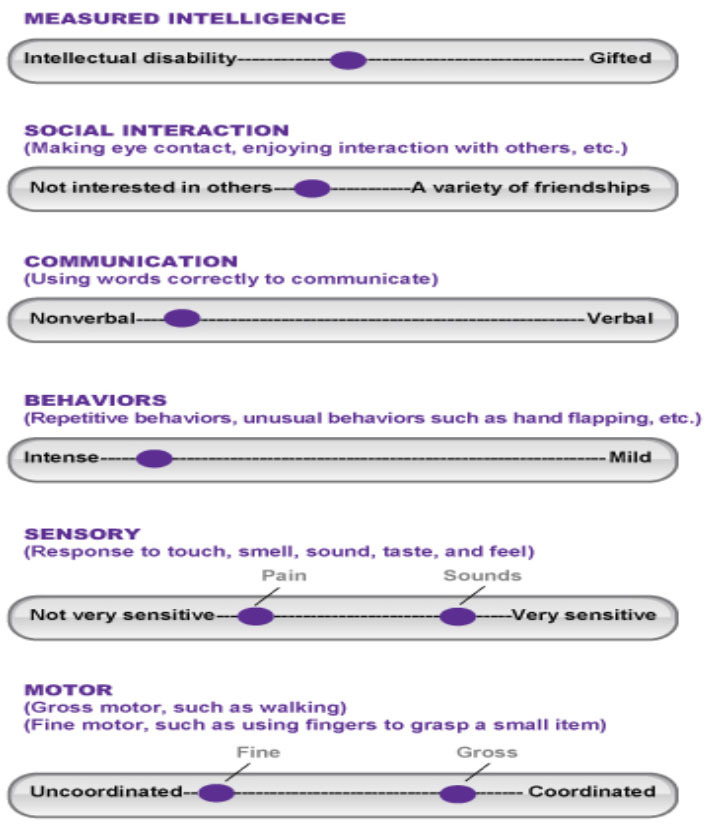
	May be between intellectually disabled–gifted.
	May have no interest in others or interest in a variety of friendships.
	May be non-verbal or verbal.
	May show obvious and intense behaviors or repetition may be mild.
	May be insensitive overly sensitive to sound, smells, pain, etc.
	May be uncoordinated or coordinated.

The Centers for Disease Control in the USA indicates that ASD affects one in 59 children (2). Globally, one out of every 160 children is diagnosed with ASD (3). Even though the prevalence of ASD is harder to determine in low- and middle-income countries, there are some researchers that have documented that the prevalence of autism is growing (4–7).

Many of the challenges in documenting ASD in developing countries are in the different presentations of symptoms as well as how typical symptoms are interpreted. For example, a common sensory sensitivity to shirt tags in clothes worn by US children may have no comparison to African children's clothing where no tag is present. Or that there is only one shirt to wear does not provide the child the option of preferred shirt selection. Likewise, limited food options may account for the limited food sensitivities. In another example, hand flapping may be interpreted as a possession by a spirit in the African context. Given that the majority of knowledge generated about ASD comes from high-income countries, such as the United States (8), there is a need to better understand cultural differences in the identification of ASD so as to develop effective screening and treatment procedures that are relevant and palatable to different parts of the world (9).

In most of the African context, public education about ASD is low. The healthcare approach relies on traditional healing methods involving physiological, spiritual, cosmological, ecological, and social forces that are informed by local customs. Research shows that a lack of public education increases the risk of misinformation about the presence of ASD. Furthermore, there is a gap in the knowledge of professionals responsible for diagnostic procedures and management of ASD in Africa. Researchers routinely indicate methodological weakness in studies using African participants as well as a lack of culturally relevant phenotyping tools (8, 10–12). A more global orientation to ASD is needed to transfer the knowledge to professionals in different regions and cultures. In sub-Saharan Africa, children with ASD are diagnosed later than children in the USA, where those from impoverished regions are most impacted due to lack of awareness, stigma, and lack of professionals (4, 7). Creating awareness and training educators and healthcare workers about ASD is a pressing need for the region (6, 13, 14).

## Ethiopian Context

Ethiopia is sub-Saharan Africa's oldest nation with a 3,000-year history and an estimated population of 101 million. The country is home to diverse cultures, where 80 languages are spoken. Eight-five percent of the population lives in rural areas where access to health care and public health education is limited or there is none at all. In Ethiopian society, health is generally viewed in a traditional light, meaning that a healthy person is thought to be in a state of equilibrium among the physiological, spiritual, cosmological, ecological, and social forces (15). Knowledge about ASD in Ethiopia is relatively low across the general public, education and social sectors, and health and government officials (8, 16, 17). Without much structure, there is little awareness or mobilization of the nation's efforts to promote autism and other disabilities through intervention programs among its citizens.

Ethiopian healthcare professionals are some of the key individuals who may detect ASD, so they are an integral part of early identification and treatment. Similar to other least developed nations, however, these professionals are also still in the initial steps of understanding autism and its treatments (18). Steeped in culture, these Ethiopian providers have not yet sorted cultural assumptions from professional practices. Research in sub-Sharan countries shows that knowledge and awareness of ASD is inadequate and there is a need for education of healthcare workers to help raise the public's levels of awareness. For example, Bakare et al. (13) found that a little more than half of the participants believed that natural causes (e.g., wind, rain, etc.) could explain ASD, and this group of individuals was most likely to recommend orthodox aid (e.g., voyage to a spiritual healer) as the method to help families relieve ASD symptoms in their children. It was also found that healthcare workers tend to recognize ASD symptomatology as a result of natural causes. Of the total healthcare workers in this study, 55% believed that ASD is treatable, and 32% believed ASD to be preventable (they subscribed to intrauterine infection or supernatural forces from past sins as controllable causes). Notably, it was also found that those involved in the management of children with ASD were more likely to believe that autism is treatable and manageable.

Changing the beliefs that some healthcare workers hold about ASD could potentially foster positive help-seeking behaviors of families. To that end, specialized training, informed by science and culturally sensitive information, is needed for healthcare workers in Africa to address ASD (7, 13, 17). However, many barriers are evident. First, it is important to note that half of the population of Ethiopia is children. This is due to the nation's high birth rate [43 births/1,000 people; (19, 20)]. Second, the most common diseases children suffer from in Ethiopia are measles and malaria, diarrhea, dehydration, pneumonia, otitis media, intestinal parasites, and skin infections (19, 21). The majority of the nation's health budget is allocated to treat these infectious communicable diseases with only 2% of the budget allocated for mental health services, even though 12% of the nation's population suffers from mental illness (22, 23). Third, beyond the sources mentioned, access to mental health services above is scarce, expensive, and improbable given the remote, sometimes inaccessible, locations where families reside. Yet, this lack of access to services is likely to result in the mistreatment of children ranging from inadequate care to being rejected by regular public schools outright; because special education services are available only in a few special schools, few children have access to these services (6, 7, 24).

Addressing mental illness and developmental disabilities, such as autism, from a scientific vantage point is only a recent development in Ethiopia. Recently, there has been a promising rise in mental health awareness and an effort in promoting the educator's and health professional's knowledge in autism in particular (6, 17, 25). New efforts include attempts to integrate mental health in the primary healthcare system and graduate-level training in several universities and across the areas of psychology, social work, and psychiatry (26). However, the limited number of trained helping professionals continues to leave the country as ill-equipped and ill-prepared to provide culturally responsive and evidence-based treatment to large segments of individuals on the spectrum. The problem of a lack of mental health training programs is underscored by the population calling for and needing services, “In [the] predominantly rural area of Ethiopia alone, mental illness comprises 11% of the total burden of diseases, with schizophrenia and depression constituting the top ten of most burdensome conditions, out-ranking HIV/AIDS” [(27), p. 9].

In general, the development of university professional practice programs in the helping professions within the higher education system is still very minimal (17). Not only is education and training lacking, but also university faculty researchers have not yet taken up these efforts for measurement or their service to the community. Considering the country's socioeconomic circumstance, cultural factors (e.g., collectivism practice), and the attitudes toward mental illness, the university community should be an essential resource for leading the development of mental health care for the public good in Ethiopia (17). Taken altogether, it is clear that a commitment to high-quality and sustained professional development training for existing helping professionals (e.g., counselors, psychologists, social workers) in all systems (e.g., government, community, individual) is the required framework to enhance scientific base knowledge and culturally responsive practice in Ethiopia.

## Autism Spectrum Disorders: Nature and Treatment

In the United States, the dominant discourse surrounding the causes of ASD includes the role of genetics (genetics in response to environmental input) and necessarily excludes spiritual explanations. Much research has supported the presence of a genetic component to the complex etiology of autism, though the extent to which genes influence the development of ASD varies in the literature (14, 28–31). As far back as the 1980s, a study suggested that mental health professionals in the United States often attribute infantile autism to genetics, even more so than European mental health professionals (32). In the same vein, a recent study investigating the beliefs of the general public and childcare providers about ASD found that participants believed autism to manifest from neurological and genetic causes (33). These researchers also found that many individuals get their information about ASD from the mass media, and while most participants relayed an accurate understanding of autism, 5% noted diet and 10% noted vaccinations as the causes of autism, which is concerning given that these causes have been completely debunked (33). In considering environmental factors, it is not being argued here that the environment at home causes autism; instead, we showcase how altering dynamics in the home environment may lead to some improvements in behavior (34, 35). In short, the dichotomy between genetic or environment contribution (e.g., nature vs. nurture) regarding the *cause* of disorder as compared to an interactionist perspective regarding the *treatment* of the disorder needs to be distinguished (36, 37). This clarification may allow for a more in-depth understanding of ASD, which hopefully can lead to more support from the family unit toward functioning as harmoniously as possible.

### Treatment in Africa

It is worth re-emphasizing that the diversity of the cultures in Africa contributes to different conceptualizations about the cause and treatment for ASD in children (38). The various conceptualizations of symptoms require a culturally sensitive diagnostic procedure and treatment protocol. Clinicians are required a nuanced understanding of the parents' experiences and cultural beliefs so as to be able to attend to the presence of symptoms even when these are presented in a manner that may differ from the dominant descriptions of ASD in the US literature. For example, symptoms of ASD such as self-stimulating behaviors or rigid adherence to routines may be characterized as evidence of being possessed by parents. Rituals may be repetitive stereotypic or understandable acts used to ward off possessions (e.g., touching a stick blessed by a healer planted outside the door). Skilled clinicians need to be able to identify symptoms and not dismiss concerns as irrelevant.

A US evaluation process may be unfamiliar and unsuccessful. For instance, de Vries (8) summarizes Berg (10) and points out that filling out a “yes or no” questionnaire about a child may be a foreign concept to many families in Africa, as an oral, or story-telling, tradition would be more culturally appropriate. Similarly, African children regularly wait 18 months for a basic diagnostic assessment, and there is a notable delay between the parents' first concerns and diagnosis (8, 39, 40). Therefore, long delays before seeking services may not be particularly concerning for parents. When parents do seek help, the majority of parents were seeking a “cure” for their child's condition (41). This is important to note because there is currently no cure for ASD, but managing symptoms and building life skills can be helpful.

Once a challenge is identified, there are limited services in Africa that can provide psycho-education and referral to specialized schools (8). Given this lack of access to trained professionals, it is typical for parents or caregivers to provide interventions for ASD management (8). As such, a multidisciplinary approach to treating ASD is uncommon and not concerning to African parents of children with special needs or developmental disorders (13). Without a firm understanding of the processes of treatment (e.g., modeling, re-enforcement, and practice), one parent wondered why they needed to attend so often when “they just do the same thing every day we come, it's never different.” This reality has led researchers to assert that “no African countries have policies or good practice guidelines for assessment, treatment, education, and adult support of individuals with ASD” [(8), p. 131; (42)].

One of the cornerstones of mental health treatment in Africa involves consulting traditional healers, which differs from the Western understanding of treatment or management of psychological health concerns and developmental conditions. Depending on the cultural context, individuals involved, and geographical location, treatments can vary from traditional (e.g., prayers) to more modern (e.g., including going to hospitals) and include combinations of each type of treatment (41). For example, Gona et al. (41) found that the community was beginning to view ASD as a disorder that could have cultural and medical implications, which highlights not only the need for cultural competency when working to understand treatments for ASD in Africa but also not to discount the fact that some people do value and seek out more modern treatment.

## The Current Study

The purpose of this study is to measure the effectiveness of an ASD training provided to helping professionals in Ethiopia. The training occurred as part of a 5-day workshop concerning cultural competencies in the diagnosis and treatment of anxiety, depression, posttraumatic stress disorder, schizophrenia, and ASD. In this manuscript, we address the following research questions:

1. Do educators and practitioners in Ethiopia improve their ability to (a) detect, (b) assess, and (c) plan interventions for individuals with ASDs?
2. How can helping professionals improve multicultural competencies (e.g., knowledge of community culture, personal involvement, resource and linkages, and organizational policy and procedure) to provide mental health service to individuals with different backgrounds?

## Methods

This pre–post method was guided by a quantitative approach to examine the effectiveness of professional development training on helping professionals attain autism intervention competency (e.g., awareness and knowledge).

### Participants

The sample (*n* = 34) consisted of educational and health providers (e.g., special education teachers, counselors, psychologists, therapists, psychiatric nurses) in Ethiopia who attended a 5-day training on mental health disorders. All have already met the requirements to practice in their areas of discipline. None were exposed to US training competencies during their original course of studies.

The training took place at the request of the host university in Ethiopia. The Office of Quality Assurance and Audit Directorate at the University of Gondar notified the trainees about the study and invited volunteers to participate. Trainees who did not wish to participate in data collection were unknown to the researchers and were not excluded from the training. Study participation was voluntary. At the start of the in-person training, the professionals were asked to sign a consent form to engage in the training and assessment process if they wish. Out of 39 participants who attended the training, 34 of them voluntarily participated in this study.

All but one of the participants reside in an urban area of Ethiopia. Twenty-three of the participants identified themselves as male, and 11 as female. Twenty-four identified their ethnic group as Amhara, whereas four listed Oromo, one Tigray, and five contained no information. Ages ranged from 23 to 39 years old. None of the participants disclosed physical disabilities. Fourteen participants hold degrees in psychology, five in nursing, seven in social work, and eight in counseling and other educational fields. Eleven individuals listed “other” as their type of degree. One-third of the individuals' highest level of education was a bachelor's degree, and two-thirds had a master's degree. The amount of time in their current positions ranged from less than one full year to 11 years in mental health service provision. The predominant occupations of individuals participating in the training were counselors, social workers, nurses, and lecturers, each making up about 20% of the sample. Other professions include psychotherapists, teachers, and special educators.

Regarding the participants' mental health training, 21 participants disclosed no previous training in ASDs. Conversely, eight reported having experience working with clients diagnosed with ASDs.

The training was offered during summer for 5 days for 8 h each day. At the beginning of the training, the outline of the training curriculum was distributed and reviewed with the participants.

## The Nature of Autism Training

The organization of the training was based on culturally responsive contemplative pedagogy and a comprehensive autism training model that includes both didactic presentation and experiential and group learning projects. A culturally responsive contemplative pedagogy is an approach that utilizes the participants' culture and regional experience in conceptualizing the Westernized definition of symptoms and intervention. For example, the participants provided case examples from their lived experience, and a comparison was made to the criteria and interpretation of the criteria based on DSM-V. The training was designed to be delivered through forethought, performance, and self-reflection. At the beginning of the training, trainees had been engaged in classroom activity and discussion that monitor their level of motivation and explore their fear and expectation to take the training. During the training, participants were engaged in experiential learning that involves role playing, case demonstrations, and case analysis. Efforts were made to utilize culturally and contextually relevant cases and demonstration. At the end of the training, participants were engaged in self-reflection of their learning process.

The overall goal of the training was to enhance participants' understanding of ASDs. Topics range from characteristics of individuals with ASDs, history of ASDs and various theories of autism, systems and institutions involved in the diagnosis, treatment, case management of children with ASDs, and use of functional behavior assessment for program planning, as well as identifying evidence-based culturally responsive treatment methods for individuals with ASDs.

### Instrumentation

This study was a single-group, pre–posttest design. The following instruments are used to collect data: (a) self-created pre- and posttraining ASD knowledge assessment test, (b) the Cultural Competence Self-Assessment Questionnaire (CCSAQ), and (c) demographic information including age, gender, residential area, ethnicity, job, educational background, and training and professional experiences. A summary is presented below.

The *ASD knowledge assessment test* was constructed with 15 items that test the participants' knowledge about symptoms of ASDs, diagnostic information, history of the disorder, cause and prevalence, intervention, and treatment. A higher score of the test indicates the trainees' full understanding of the disorder. The test was administrated before and after the trainees received the training. The total score on the ASD knowledge assessment test measures the knowledge of symptoms, identification, nature/expression of impairments, cause, and interventions.

The *CCSAQ* is a standardized assessment tool developed by the Portland Research and Training Center in 1995 (43). The CCSAQ is based on the Child and Adolescent Service System Program's cultural competence model (44), which describes competency in terms of four dimensions: attitude, practice, policy, and structure. The CCSAQ has proven useful in various organizations around the USA. We used the CCSAQ service provider version with minor modification to fit into the Ethiopian context. For example, Ethiopia is a multiethnic country, which means a person with a specific ethnic group could be a minority in one region, but could be part of the majority group in another region. Hence, we utilize a contextual definition of individuals with minority backgrounds instead of the word community of color. The CCSAQ is a 56-question self-report with four subscales: community knowledge, personal involvement, resource and linkage, and policy and procedures. The CCSAQ uses a four-point Likert scale with responses ranging from “not at all” to “very well.” The CCSAQ subscales have yielded alpha coefficients ranging from 0.60 to 0.80.

### Data Collection Procedure

Once consent was obtained, all participants completed a demographics questionnaire and the Cultural Competence Self-Assessment Questionnaire. All participants then attended a 5-day workshop on the assessment practices and interventions for depression, anxiety, schizophrenia, posttraumatic disorder, and ASD. The ASD portion of the training was covered over the course of 2 days. Before the first lesson of each disorder, the participants completed a pretest of their knowledge on that specific disorder. Then, the researchers provided education for the participants on the symptoms and evidence-based interventions associated with ASDs and the cultural responsiveness and competency approach for assessment and intervention.

The training was conducted mostly in English, as the university is designated as an English-medium location, meaning classes are to be taught in English. However, Amharic was also used during the role-play activities and at times to allow attendees to express themselves best through a more familiar and fluent language. The lead author spoke Amharic as her native language and was present during the training. At the end of the training, the participants took a posttest to measure their knowledge of ASDs. The pre- and posttests were the same test administered at two separate times to measure learning.

### Ethical Considerations

A US university's IRB committee approved the study. The nature and scope of the research were explained to each participant before the training. Trainees who are voluntary participants complete the demographics survey and the questionnaires for the study. Participants were informed that their participation is optional, and each of them was assured that there would be no retribution for the study's withdrawal at any time. All participants had the opportunity to ask questions before the survey and before the pre-training test was administered. They were also advised that any information provided would remain confidential.

### Data Analysis and Results

Once data were collected, these were entered into SPSS 23. In cases where it made a conceptual sense, data were prescreened to identify any outliers and missing data and to collapse the response categories of the independent variables with a smaller size. For example, in the original data, for the variable field of study, the types “counseling” and “education and other related” were combined into one category “counseling, education and other related.”

The study's main independent variables are the demographic variables presented in Table 1, which are gender and field of studies. The dependent variables are the level of ASD knowledge measured by the pre- and post-training tests and cultural competency measured by the CCSAQ. Inferential statistics were used to examine the effectiveness of the professional development training in participants' knowledge of ASD. The aggregated CCSAQ scores' means are computed for each item and each subscale.

**Table 1:** Sample demographics.

		***N***	**%**
Sex of the respondent	Male	23	68
	Female	11	32
Ethnicity	Amhara	24	70
	Oromo	4	12
	Tigray	1	3
	Not-identified	5	15
Age range	21–30	20	60
	31–40	14	40
Education	Bachelors	10	29
	Masters	23	68
	Post Masters	1	3
Field of Study	Psychology	14	40
	Social work	7	21
	Psychiatric nursing	5	15
	Counseling/other	8	24
Residentials area	Urban	33	97
	Rural	1	3
Previous training on ASDs	Yes	5	15
	No	29	85

### Participants' Understanding of Autism Spectrum Disorders

To determine the effectiveness of the training on participants' understanding of ASD, the pretest and posttest comparisons were made using paired sample *t*-tests that were conducted to compare participants' responses in four domains: symptoms, nature, cause, and interventions (see Table 2).

**Table 2:** Pre-post comparisons.

**Domain**	**Pre-training result** ***N*** **=** **34**		**Post-training result** ***N*** **=** **34**		***t*-test**
	**M**	**SD**	**M**	**SD**	
Symptoms	2.21	1.08	4.60	0.78	11.1^*^
Nature	2.09	1.15	4.63	0.4	9.459^*^
Cause	0.869	0.6255	1.78	0.42	6.552^*^
Intervention/Treatment	1.36	0.82	2.60	0.53	7.4^*^

* *p <0.01*.

**Table 3:** Participants' response on multicultural resources and use of linkages.

	***N***	**M**	**SD**
Does your agency have linkages with institutions of higher education (e.g., colleges, universities, or professional schools) that could provide you with accurate information concerning different cultural group?	34	2.5	0.96
Does your agency publish or assist in the publication of information focusing on mental health of different cultural group?	34	1.7	0.65
Has your agency conducted or participated in a needs assessment utilizing providers in communities as respondents	34	1.5	0.72
Does agency staff routinely share practice-based “success stories” involving people with minority background?	34	2.0	0.88
Has your agency conducted or participated in a research that focused about individuals with minority background	34	1.5	0.78
Does staff utilize cultural consultants who can help them work more effectively within a cultural context?	34	2.3	0.88
Does your agency convene or reward activities that promote learning new languages relevant to the communities with minority background that the agency serves?	34	1.6	0.76
Does your agency staff routinely discuss barriers to working across cultures?	34	2.4	0.96
Does your agency compile books or culturally-related written materials regarding people of culture?	34	1.9	0.89

**Figure 1:**
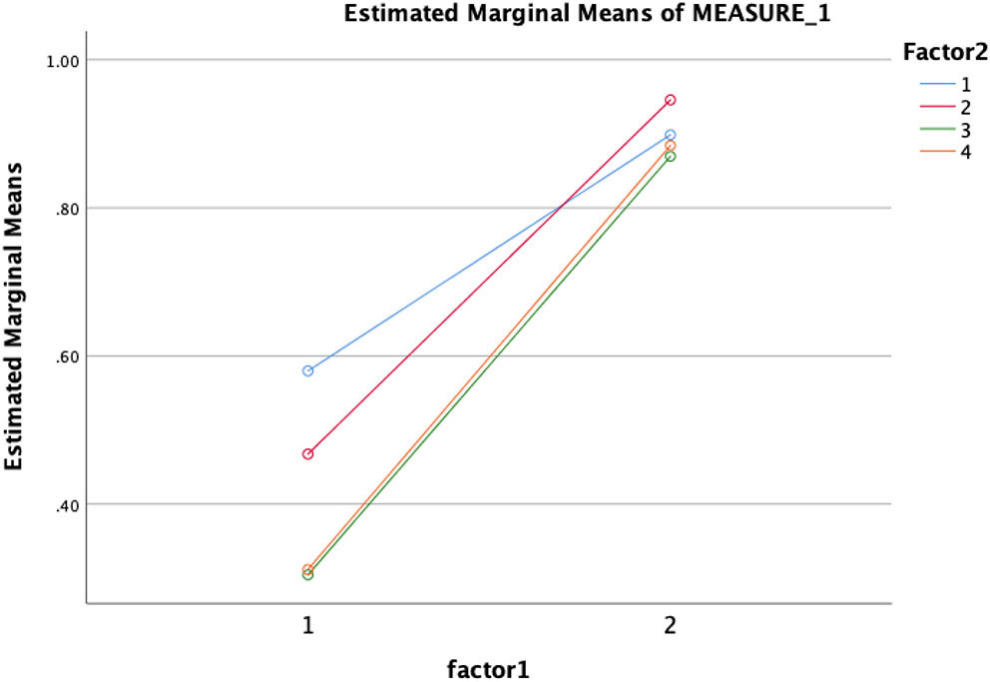
Multivariate tests.

Results showed significant improvements in learning. Specifically, there were significant gains in understanding symptoms when comparing pretest (*M* = 2.2, SD = 1) and posttest (*M* = 4.6, SD = 0.78) results: *t*_(32)_ = −11.10 (*p* < 0.01); understanding of the nature of ASDs (i.e., history, prevalence, and characteristics) before (*M* = 2.0, SD = 1.15) and after the training (*M* = 4.7, SD = 0.49): *t*_(32)_ = −9.46 (*p* < 0.01); understanding the cause and associated factors of ASDs before (*M* = 0.87, SD = 0.63) and after the test (*M* = 1.8, SD = 0.42; *t*_(32)_ = −6.6 (*p* < 0.01); and preparedness to use evidence-based intervention and treatment when comparing before (*M* = 1.36, SD = 0.82) and after (*M* = 2.6, SD = 0.53); *t*_(32)_ = −7.4 (*p* < 0.01) training scores. A multivariate analysis by group (age, gender, field of study, and experience) showed (see Figure 1) that there was a significant change in all four variables from pretest to posttest, which means participants' understanding about ASD is increased as a result of the in-service training.

### Assessment of Culturally Informed Services and Practices Among Participants

Using a Likert-type scale (i.e., from 1 = not at all, to 4 = very often), participants responded to how accurately the statement described their cultural responsiveness in four subscale areas that includes knowledge of the community, personal involvement, resources and linkage, and organizational policies and procedures.

**Figure 2:**
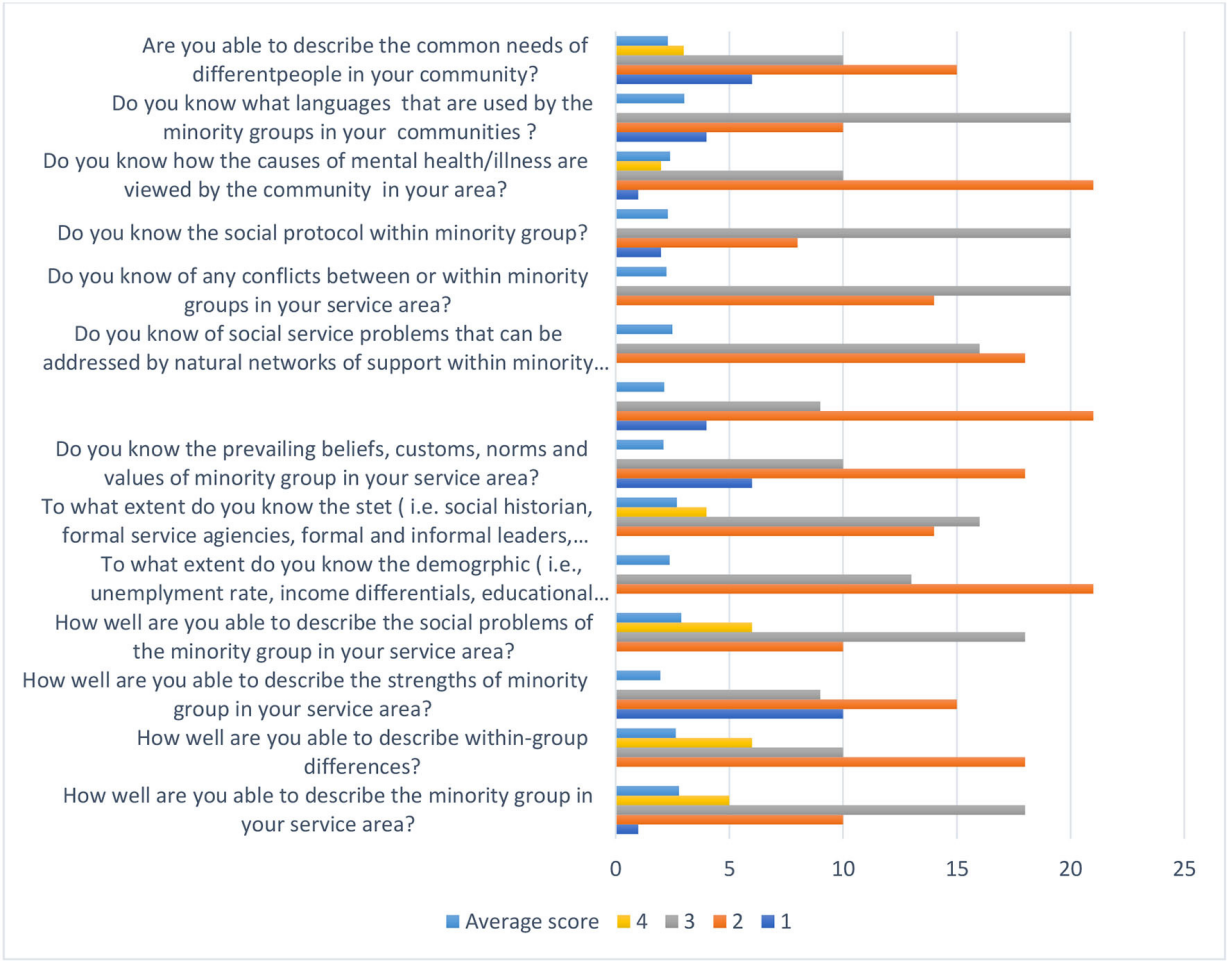
Participants knowledge of the minority communities they serve.

The subscale *participants' knowledge of the minority communities* they serve is described in Figure 2. The aggregated mean shows that participants scored above average for eight of the 14 items. These include knowing which *languages are used by the minority groups* in your communities (*M* = 3.02, SD = 1.02), being able *to describe the minority group* in your service area (*M* = 2.79, SD = 0.94), identification of *within-group differences* (*M* = 2.69, SD = 0.98), information about the *social problems* of the minority groups within your service area (*M* = 2.89, SD = 0.88), identifying minority group (i.e., social historian, formal service agencies, formal and informal leaders, advocates, clergy or spiritualists) (*M* = 2.7, SD = 1.09), awareness of *conflicts between or within minority groups* (*M* = 2.24, SD = 0.86), awareness of the *social protocols* required within communities (*M* = 2.3, SD = 0.89), and familiarity of how the *causes of mental health/illness are viewed* by the community in your area (*M* = 2.4, SD = 0.86). However, participants scored below average on geographic *demographic information* (i.e., unemployment rates, income differentials, educational attainment, crime rate, birth/death rate, homicide rates) within the minority groups they serve (*M* = 1.9, SD = 0.88); ability to describe the *common needs* of different people in your community (*M* = 1.97, SD = 0.86); awareness of the *prevailing beliefs, customs, norms, and values* of the minority group in your service area (*M* = 2, SD = 0.65); knowledge of the *social service needs that go unaddressed* by the formal social service system (*M* = 2.01, SD = 0.94); knowledge of *social service problems that can be addressed* by natural networks of support within the minority group (*M* = 2.03, SD = 0.94); and the *prevailing beliefs, customs, norms, and values* of the individuals from minority ethnic backgrounds in their service area (*M* = 2.0, SD = 0.96).

**Figure 3:**
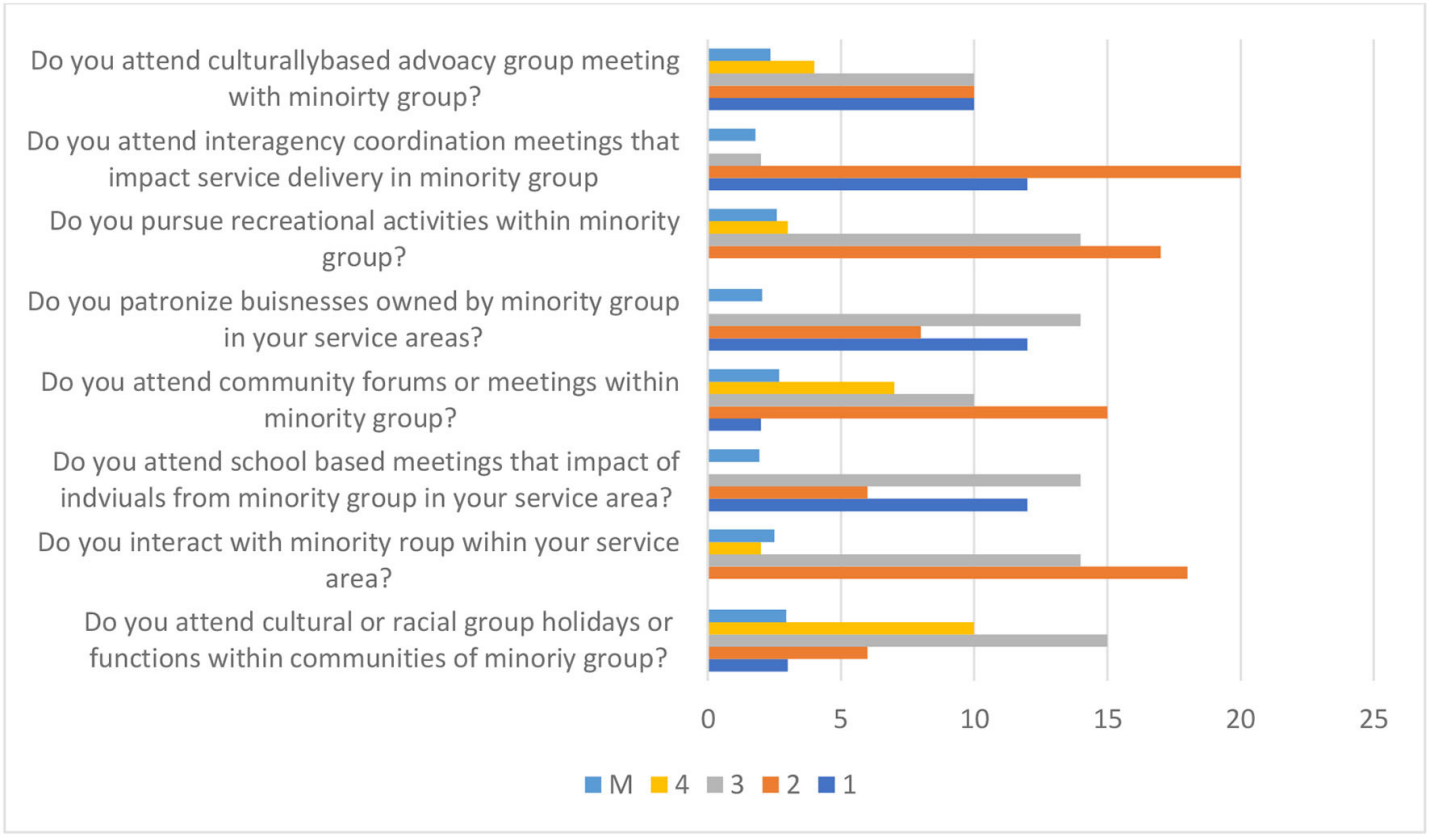
Participants score on personal involvement in diversity.

The *personal involvement* subscale asks eight items to rate participants' involvement in different cultural contexts with the communities they serve from minority backgrounds (see Figure 3). Descriptive analysis of personal involvement shows that, on average, participants indicate a high level of time spent *working and meeting others* from different cultural backgrounds (*M* = 3.0, SD = 0.88). Participants also scored above average for items that include the following: *participation to community forums* (*M* = 2.68, SD = 0.98), cultural or ethnic-based *holidays* (*M* = 2.35, SD = 0.98), pursue *recreational activities* (*M* = 2.59, SD = 1.00), *patronizing businesses* owned by a minority group (*M* = 2.59, SD = 0.98), and *interactions* with minority groups in their service area (*M* = 2.5, SD = 0.96). All of these indicate that professionals spent a moderate level of time working and meeting others from different cultural backgrounds than their own. Participants' responses found to be below average include attendance at *interagency coordination meetings* (*M* = 1.79, SD = 0.86) and *school-based meetings* (*M* = 1.94, SD = 0.88), which indicates participants' low level of personal involvement in coordination with community-wide initiatives.

The aggregated score for the *multicultural resources and use of linkages* subscale (see Table 4) indicates that professionals in the study lack knowledge of resources and relevant links to support culturally competent service provision. Participants rated below average for knowledge of community agencies' *publications* (*M* = 1.7, SD = 0.65), the conducting of a *need assessment* (*M* = 1.5, SD = 0.72), conducting *research* (*M* = 1.5, SD = 0.78), promoting *new language learning* (*M* = 1.6, SD = 0.76), and being able to compile books *or culturally related written materials* regarding people of cultures outside their own (*M* = 1.9, SD = 0.89). Participant scores on the following items were above average: connections with *institutions of higher education* (*M* = 2.5, SD = 0.96), routine discussion with their staff about *barriers working across cultures* (*M* = 2.4, SD = 0.96), staff's ability to utilize relevant *cultural consultants* (*M* = 2.3, SD = 0.88), and staff routinely sharing practice-based “*success stories*” (*M* = 2.00, SD = 0.88).

**Table 4:** Agency policies and procedures addressing cultural diversity.

**As a matter of formal policy, does your agency**	***N***	**M**	**SD**
• Use culture-specific assessment instruments for diagnosis?	34	2.5	0.89
• Use culture specific treatment approaches?	34	2.5	0.88
• Envision community empowerment as a treatment goal?	34	2.3	0.89
• Review case practice on a regular basis to determine relevancy to clients with minority background?	34	1.5	0.78
• Provide or facilitate child care?	34	1.2	0.96
• Consider culture in service plans?	34	1.9	0.88
• Conduct outreach to community based organizations, social service agencies, natural helpers, or extended families?	34	1.7	0.72
• Take referrals from non-traditional sources?	34	2.6	0.94
• Translate agency materials into languages that reflect the linguistic diversity in your service area.	34	1.5	0.66
• Advocate for a better quality of life for minority group in addition to providing services.	34	2.1	0.76
Is information on the ethnicity or culture of clients specifically recorded in your organization management information system?	34	2.7	0.88
Does your organization or agency reach out to churches and other places of worship, clergy persons, ministerial alliances, or indigenous religious leaders in communities with minority background?	34	1.9	0.89

The use of *organizational policy and procedures* to promote diversity and culturally responsive practices subscale (see Table 5) indicates that participants' responses are above average for agency policy and procedures on items including the following: use of cultural specific assessment (*M* = 2.5, SD = 0.89), use of cultural specific treatment approaches (*M* = 2.5, SD = 0.88), *e*nvisioning community empowerment as a treatment goal (*M* = 2.3, SD = 0.89), and taking a referral from a non-traditional source (*M* = 2.6, SD = 0.94). However, participants' responses were below average on the policy and procedure practice to review cases on a regular basis to ensure they are sensitive to clients with a minority background (*M* = 1.5, SD = 0.78); provide or facilitate child care (*M* = 1.2, SD = 0.96); consider culture in service plans (*M* = 1.9, SD = 0.88); use outreach to community-based organizations, social service agencies, natural helpers, or extended families (*M* = 1.7, SD = 0.72); and translate agency materials into languages that reflect the linguistic diversity in your service area (*M* = 1.5, SD = 0.66). Regarding the establishment of agency procedures for *r*ecording information on ethnicity or cultural clients, participants' score was above average (*M* = 2.7, SD = 0.88). The agencies' procedures were rated below average (*M* = 1.9, SD = 0.89) for reaching out to church and other places of worship or indigenous religious leaders in communities of the minority.

**Table 5:** Descriptive score of each subscale by gender.

**Subscale**	**Total** **(*****N*** **=** **34)**		**Women** **(*****N*** **=** **7)**		**Men** **(*****N*** **=** **27)**	
	**M**	**SD**				
Community knowledge	2.620	0.4356	2.7737	0.33902	2.4670	0.44067
Personal involvement	2.381	0.62131	2.433	0.70238	2.3333	0.61017
Resources linkages	1.933	0.61273	1.8024	0.87646	1.9904	0.52932
Organizational policy	2.03	0.63049	1.9857	0.56988	2.0936	0.65353

Overall, participants scored above average on community knowledge (*M* = 2.62, SD = 0.435) and personal involvement (*M* = 2.38, SD = 0.621) when compared to resource use and linkages (*M* = 1.93, SD = 0.61) and organizational policies (*M* = 2.03, SD = 0.63). Participants' responses for the four subscales based on gender (Table 5) show that female participants, on average, score slightly higher compared with the men's responses on community knowledge (female: *M* = 2.77, SD = 0.33 | male: *M* = 2.4670, SD = 0.44) and personal involvement (female: *M* = 2.43, SD = 0.702 | male: *M* = 2.33, SD = 0.610). However, men's responses for knowledge of resource linkage (*M* = 1.99, SD = 0.52) and organizational policy and procedures (*M* = 2.09, SD = 0.65) are slightly higher than women's (*M* = 1.80, SD = 0.87 for resource and linkage; *M* = 1.98, SD = 0.569 for organizational policy).

## Discussion and Future Directions

Much of the literature regarding professional development stresses the importance of designing activities that are compatible with the characteristics of adult learners (45). Adult learners tend to be self-directed in that they can identify their weaknesses and they can be partners in developing corrective plans of action. Because this 5-day workshop was at the request of local health service providers and conducted in conjunction with university support, it does suggest that these professionals were indicating a readiness for learning. Under this assumption, most workshop trainings focus on the criteria of professional satisfaction to evaluate the usefulness of the presentations. While this is arguably important, it is also sorely inadequate to measure the effectiveness of the training activities. Indeed, Moore (45) also cautions that one of the challenges of training adults is their tendency to adhere to their preferred ways of knowing and relying on previously established information. As such, it was critical to measure the learning of these participants in a manner that would be expected in preparation for a professional exam. Specifically, the study sought to determine the effectiveness of the workshop by evaluating changes in Ethiopian professional's ability to (a) detect, (b) assess, and (c) plan interventions for individuals with ASDs. Results showed uniformly significant improvements in learning in all areas.

It is perhaps not surprising that bright individuals would learn quickly. However, given that these professionals had very limited training in ASD, and during the training, as noted by Moore (45), they showed patterns where they relied on, and at times had interference from, traditional interpretations for the causes of ASD, it is important and impressive to note their adherence to decision-making that was scientifically informed during the posttest. Identifying the correct answer during a recognition task (i.e., multiple choice where the answer is available) may have provided an opportunity for good guessing. However, phony learning is much harder to pull off when the task is in the conceptualization of the practice cases.

Regardless of the professional development topic, it is essential that all education and training experiences explicitly address how professionals need to relate to individuals from diverse cultural and linguistic backgrounds. Training needs to also explicitly address the implementation steps for developing any new practices. As such, we also considered the multicultural competencies (e.g., knowledge of community culture, personal involvement, resource and linkages, and organizational policy and procedure) of these helping professionals. Here, the data were more mixed; yet, they highlight key areas for development. For example, under the *knowledge of community culture*, there was room for improving the *common needs* of different people in your community; awareness of the *prevailing beliefs, customs, norms, and values* of the minority group in your service area; knowledge of the *social service needs that go unaddressed* by the formal social service system; knowledge of *social service problems that can be addressed* by natural networks of support within the minority group; and the *prevailing beliefs, customs, norms, and values* of these groups. In fact, there were areas of improvement noted across all of the subscales (i.e., *personal involvement, resource and linkages*, and *organizational policy and procedure*). In practical terms, these data can help these professionals identify areas of improvement in their practice. The areas of growth can serve as a checklist for them in their planning for the next steps in professional practices.

As with all studies, this one has a number of limitations. First, the sample size was small. This study only looked at immediate knowledge and skill acquisition. There was no follow-up to see if information was retained and used in subsequent practice. The training used vignettes and was not with real patients; as such, there was limited decision-making not confounded by the interpersonal interactions between the provider and the patient.

## Conclusions

It is likely that these professionals would further benefit from repeated professional practice and coaching during *in situ* case decisions. Given the growing awareness of ASD, there is a clear need to expand the education efforts across Africa and other developing countries. Kegan (46) provides helpful guideposts regarding the way to create a bridge from current beliefs and practices to new directions in professional service provision. The process includes the following:

- Asking professional what they already know about—or want to know about—related to the mental health professional development topic.
- Surveying professionals regarding both successes and challenges on the topic prior to the teacher professional development learning events.
- Inviting professionals to share stories from their experiences—as students or when they have obtained their professional role—that connect with the professional with the new topic.

This type of iterative and reflective process can ensure that the next steps in professional development planning are accomplished. These steps also serve to develop and sustain professional motivation.

The results from this study can serve as one data point in the effort to describe the status of the needs for mental health training for service providers in Ethiopia. By beginning to document benchmarks regarding the use and understanding of US mental health standards, we hope to be able to offer an avenue to blend traditional and modern practices that resonate with the local community [see Hughes et al. (47)]. That is, although there is a general public perception and acceptance that there is a need to expand services to the African population, there is little to document the current state of the need.

## Data Availability Statement

The raw data supporting the conclusions of this article will be made available by the authors, without undue reservation.

## Ethics Statement

The studies involving human participants were reviewed and approved by Duquense University IRB. The patients/participants provided their written informed consent to participate in this study.

## Author Contributions

WZ and TH design the study, conduct the training, collect the data, and write the result. GK provided consultation on the statistical analysis of the data. All authors contributed to the article and approved the submitted version.

## Conflict of Interest

The authors declare that the research was conducted in the absence of any commercial or financial relationships that could be construed as a potential conflict of interest.
